# Integrated prognostication of intrahepatic cholangiocarcinoma by contrast-enhanced computed tomography: the adjunct yield of radiomics

**DOI:** 10.1007/s00261-021-03183-9

**Published:** 2021-06-24

**Authors:** Mario Silva, Michele Maddalo, Eleonora Leoni, Sara Giuliotti, Gianluca Milanese, Caterina Ghetti, Elisabetta Biasini, Massimo De Filippo, Gabriele Missale, Nicola Sverzellati

**Affiliations:** 1 Department of Medicine and Surgery (DiMeC), University of Parma, Via Gramsci 14, Parma, Italy; 2 Unit of “Scienze Radiologiche”, University Hospital of Parma, Parma, Italy; 3 Servizio Di Fisica Sanitaria, University Hospital of Parma, Parma, Italy; 4 Unit of Radiology, University Hospital of Parma, Parma, Italy; 5 Unit of Infectious Diseases and Hepatology, University Hospital of Parma, Parma, Italy

**Keywords:** Survival, Intrahepatic cholangiocarcinoma, Multidetector Computed Tomography, Radiomics, Interobserver Variability

## Abstract

**Purpose:**

To test radiomics for prognostication of intrahepatic mass-forming cholangiocarcinoma (IMCC) and to develop a comprehensive risk model.

**Methods:**

Histologically proven IMCC (representing the full range of stages) were retrospectively analyzed by volume segmentation on baseline hepatic venous phase computed tomography (CT), by two readers with different experience (R1 and R2). Morphological CT features included: tumor size, hepatic satellite lesions, lymph node and distant metastases. Radiomic features (RF) were compared across CT protocols and readers. Univariate analysis against overall survival (OS) warranted ranking and selection of RF into radiomic signature (RSign), which was dichotomized into high and low-risk strata (RSign*). Models without and with RSign* (Model 1 and 2, respectively) were compared.

**Results:**

Among 78 patients (median follow-up 262 days, IQR 73–957), 62/78 (79%) died during the study period, 46/78 (59%) died within 1 year. Up to 10% RF showed variability across CT protocols; 37/108 (34%) RF showed variability due to manual segmentation. RSign stratified OS (univariate: HR 1.37 for R1, HR 1.28 for R2), RSign* was different between readers (R1 0.39; R2 0.57). Model 1 showed AUC 0.71, which increased in Model 2: AUC 0.81 (*p* < 0.001) and AIC 89 for R1, AUC 0.81 (*p* = 0.001) and AIC 90.2 for R2.

**Conclusion:**

The use of RF into a unified RSign score stratified OS in patients with IMCC. Dichotomized RSign* classified survival strata, its inclusion in risk models showed adjunct yield. The cut-off value of RSign* was different between readers, suggesting that the use of reference values is hampered by interobserver variability.

**Supplementary Information:**

The online version contains supplementary material available at 10.1007/s00261-021-03183-9.

## Introduction

Cholangiocarcinoma is the most common malignancy of biliary tract and the second most common primary hepatic malignancy, it accounts for 15–20% of primary hepatobiliary malignancies, mostly affecting male elderly [1], with the highest prevalence in Asian countries [2]. The anatomical distribution implies different management options: intrahepatic cholangiocarcinoma is the most frequent site of origin [3], notably the “mass‐forming” growth pattern (intrahepatic mass-forming cholangiocarcinoma, IMCC) represents over 60% of cases [4]. The American Joint Committee on Cancer (AJCC)/Union for International Cancer Control (UICC) staging system 8th edition is used for prognostic stratification and treatment choice [5]. Notably, the AJCC staging system incorporates clinical characterization of intrahepatic cholangiocarcinoma by contrast-enhanced computed tomography (CT) [6–8].

Imaging plays a pivotal role for diagnosis, staging, and prognostication of IMCC [7, 9–11]. However, visual assessment of CT and manual annotation suffer from subjective variability: this might result in variable performance in clinical management [7, 12, 13]. Quantitative analysis of CT data was reported for standardized characterization of cholangiocarcinoma by radiomic features (RF), especially for accurate prediction of lymph node metastases beyond visual morphologic criteria [14, 15]. The use of radiomics in hepatic malignancies has been thoroughly explored for non-invasive differentiation of histology or for prediction of lymph node metastases, into the scenario of the so-called soft-outcome measures [16–20]. A minority of authors challenged the use of radiomics for prediction of survival and disease-free survival [21, 22].

Accurate non-invasive prognostic descriptors of IMCC are deemed of major clinical support for personalized therapeutic approach because IMCC is one form of intrahepatic cholangiocarcinoma for surgical option, and therefore radiomics might represent a relevant complement in pre-surgical clinical management. Most of the published research focused on population selected by clinical treatment, namely surgery or embolization [23, 24]. However, the application of radiomics on broader population is still lacking, especially for management throughout the full process of treatment decision.

The aim of this study was to test radiomics for prognostication of IMCC and to develop a prognostic model that combines clinical parameters and radiomics for prediction of survival in patients with IMCC, across the full range of treatment options.

## Materials and methods

### Study population

Patients with histologically proven IMCC at *BLINDED* between January 2007 and December 2018 were retrospectively retrieved. The Institutional Review Board approved this study (Prot. 43,024) and informed consent was retrieved for enrolled patients.

Inclusion criteria were: (a) immunopathological diagnosis of IMCC; (b) age > 18 years; (c) baseline hepatic venous phase CT. Exclusion criteria were: (a) previous treatment; (b) periductal infiltrating or intraductal growing patterns; (c) missing clinical data; (d) motion artifact on CT imaging. Demographics and clinical data were collected, including histologic grading and treatment. All patients underwent contrast-enhanced CT with injection of high-concentration iodine contrast (400 mg I/mL, Iomeron 400, Bracco, Italy), volume 90–130 mL (based on patient weight), flow rate 3–4 mL/s. Contrast-enhanced scan was triggered by 150 HU density in abdominal aorta (at level of celiac axis) and portal venous phase was acquired with 60 s delay.

### Morphologic CT descriptors

Two readers (R1, radiologist with 15-year experience in abdominal imaging; R2, 4th year radiology resident) independently reviewed the CT scans (blinded to clinical and pathological information) and collected the following standard clinical parameters:

- Tumor size: maximum diameter on axial plane (manual caliper);
- Satellite hepatic lesions;
- Lymph node metastasis defined as follows:
  o Short-axis > 10 mm,
  o Central necrosis (areas of lower density),
  o Contrast enhancement compared with liver [25];
- Distant metastasis in organs other than liver or lymph nodes.

In case of disagreement, a final consensus was reached between readers for binary variables. Otherwise, discrepancy in manual caliper of tumor size were deemed substantial when exceeding 5 mm in lesions larger than 25 mm or exceeding 20% in lesions smaller than 25 mm (reference: R1 radiologist). Such discrepancy was resolved with a joint reading session. If the discrepancy was below the established threshold, the mean between the two readers was considered.

### Volumetric analysis: segmentation and extraction of radiomic features

DICOM headers were recorded to assess variability between acquisition and reconstruction parameters.

Each reader independently outlined tumor boundaries on portal venous phase by manually drawing the volume of interest (VOI) with a dedicated software (3D Slicer version 4.10.2). 108 RF were calculated by SlicerRadiomics® [26], including shape, first-order, Gray-Level-Co-occurrence-Matrix (GLCM), Gray-Level-Run-Length-Matrix (GLRLM), Gray-Level-Size-Zone-Matrix (GLSZM), Neighboring-Gray-Tone-Difference-Matrix (NGTDM) and Gray-Level-Dependence-Matrix (GLDM) features. The RF subsets obtained from segmentations of R1 and R2 were named RF-R1 and RF-R2, respectively.

### Statistical analysis

Continuous data were reported as median, first and third quartiles (interquartile range, IQR). Categorical data were reported as frequency of occurrence.

The primary outcome of this study was overall survival (OS), it was calculated as number of days between the date of CT and date of death. The last follow-up was set at 5 years and dataset lock was on October 22, 2019. Association between clinical parameters and OS was tested by Mann–Whitney *U* test or Pearson chi-square test, as appropriate.

#### Variability of radiomic features

Variability of RF across acquisition and reconstruction parameters was tested by Kruskal–Wallis test and Spearman correlation. Reconstruction algorithm settings B30s and B40s were not considered in statistical analysis because each occurred only once.

Interobserver variability of RF was tested by intraclass correlation coefficient (ICC) based on single rater, absolute-agreement, 2-way mixed-effects model. Single rater ICC was considered because machine learning models were independently developed for each segmentation. ICC values < 0.5 were deemed for high variability, 0.5–0.75 moderate, 0.75–0.9 low, and > 0.90 very low [27]. RF with high variability were excluded from prognostic modeling.

#### Stratification of risk

**Fig. 1:**
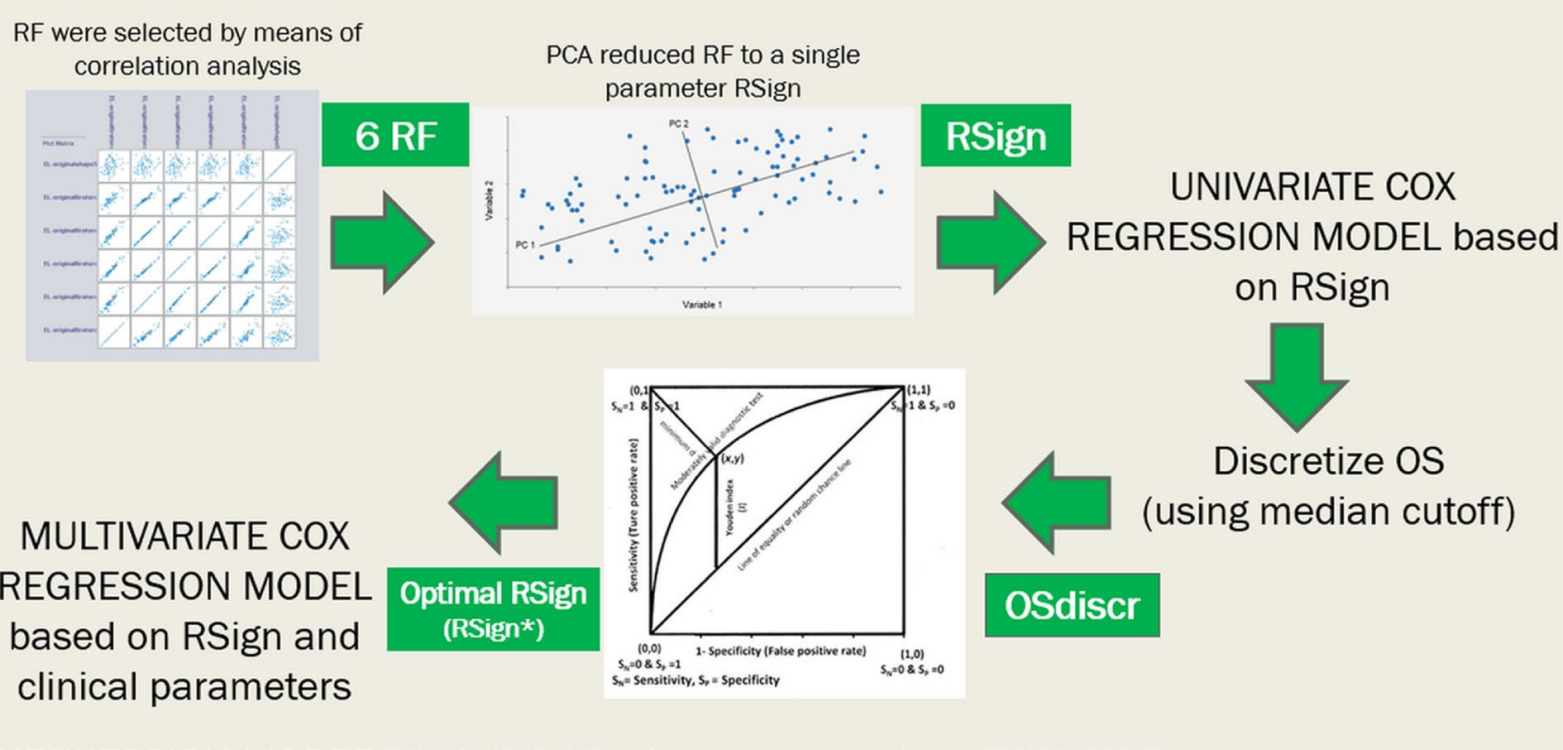
The flowchart summarizes the multistep process for selection of radiomic features (RF) and building of radiomic signature (RSign)

Multistep process for developing prognostic models is thereafter detailed (Fig. 1).

##### Radiomic signature

Pearson correlation analysis between each RF and OS were performed. RF were ranked in descending order according to their correlation coefficients for both RF-R1 and RF-R2 subsets: highly correlated RF that belonged in both readers were selected. Principal component analysis (PCA) was applied to the selected RF in order to reduce the dimensionality of radiomic predictors and to extract a single radiomic signature (RSign) for synthetized representation of all RF in a unique scale of risk. Both correlation analysis and PCA were performed by Weka v.3.8.3 [25].

Univariate Cox proportional hazards models were used to verify if RSign represented a predictor of OS. Median value of OS was used to discretize two groups, namely short-term and long-term survivors. A Receiver Operating Characteristic (ROC) analysis was performed to determine the cut-off value of RSing for optimal stratification into two risk groups: the value that yielded the largest vertical distance between the ROC curve and the random chance line (Youden index) was chosen as optimal cut-off for RSign (RSign*). Kaplan–Meier survival curves for the two risk groups were calculated and then compared using log-rank test.

##### Risk models

Cox proportional hazards models were developed to evaluate RSign and clinical parameters (including morphological CT descriptors) as predictors of OS. Predictors with p-values > 0.10 were excluded from subsequent final models. Significant variables with potential confounding effect in clinical application were identified and excluded from final analysis, notably a post-hoc analysis was run in a subset of resected IMCC without chemotherapy (see “Analysis restricted to resected subpopulation”, Supplementary Material). Final Cox proportional hazard models were built on predictors that were significant in univariate analysis. Statistical findings of survival analysis were validated using a bootstrap procedure using 200 random samples. Logistic regression was then performed to build models for estimating the performance improvement attributable to RSign by comparing:

- Model 1: clinical parameters including age, gender, grading, and morphologic CT descriptors.
- Model 2: clinical parameters from Model 1 and RSign.

The 1-year OS was used to discretize OS for logistic regression analysis. Logistic models were compared by area under the ROC curve (AUC) [26]. Logistic classification was performed using a tenfold cross-validation procedure. Akaike information criterion (AIC) and Likelihood Ratio Test were used for models comparison [28].

Statistical analysis was performed by SPSS Statistics 23(IBM Corp., Armonk, N.Y., USA) and R 4.0.2 (http://www.R-project.org) [29].

## Results

Seventy-eight patients (age range 35–89 years, 43 men) were selected, median follow-up was 262 days (IQR 73–957) (Table 1). 62/78 (79%) patients died, notably 46/78 (59%) within 1 year since CT. Survival data were right-censored for 16/78 (20.5%) patients, at time of dataset lock.

**Table 1:** Demographics, clinical data, morphological CT descriptors, RF are reported

Population characteristics	
*Demographics*	
Gender (male)	
Male	43 (55)
Female	35 (45)
Age (years)	61 [68–74]
Death	62 (79)
Overall survival	262 [73–957]
Survivors at 1 year	32 (41)
*Clinical data*	
Radiology	
Satellite hepatic lesion	38 (49)
Metastatic lymph node	54 (69)
Distant metastasis	25 (32)
Grading	
G1	1 (1)
G2	29 (37)
G3	48 (62)
Treatment	
Surgical resection^a^	31 (40)
Chemotherapy^a^	25 (32)
Radiofrequency ablation	11 (14)

RF type	RF name	R1	R2	ICC
*Selected RF and RSign*				
Shape	Surface volume ratio^b^	0.2 [0.1–0.3] (6)	0.2 [0.1–0.3] (6)	0.54
First order	Median^b^	70.0 [59.4–89.8] (3)	70.0 [60.0–89.8] (1)	0.80
First order	Mean	70.7 [59.1–85.7] (4)	69.1 [59.0–87.5] (3)	0.78
First order	10' percentile^b^	36.5 [21.0–47.0] (5)	34.0 [19.3–45.3] (5)	0.70
First order	90' percentile^b^	107.5 [94.3–120.0] (1)	110.0 [97.3–125.3] (2)	0.78
First order	Root mean squared^b^	76.7 [65.–90.9] (2)	76.6 [69.4–92.3] (4)	0.77
RSign		0.23 [− 1.05–1.16]	0.54 [− 1.05–1.28]	0.79

Categorical data are reported as absolute number and relative distribution (percentage in round bracket). Continuous data are reported as median (median, first and third quartiles in square brackets). RF are reported for each of the two readers (R1 and R2), the ranking position is reported for each reader using round brackets

^a^Three patients undergoing surgery were also administered adjuvant chemotherapy

^b^Selected in the final model RSign

Standard clinical parameters from CT showed discrepancy in 7.8% maximum diameter, 7.8% satellite lesions, 10.3% lymph node metastasis, 6.4% distant metastasis; the consensus was skewed toward R1 reading (Supplementary Table 1).

### Variability of radiomic features

Variability across different acquisition protocols was significant in 5/108 (5%) for RF-R1 and in 11/108 (10%) for RF-R2 (Supplementary Table 2–3).

Variability due to segmentation was high in 37/108 (34%) RF, moderate in 44 (41%), and low in 27 (25%) (Supplementary Table 4). The 71 RF with moderate to low variability were selected for modeling process.

### Stratification of risk

#### Radiomic signature

The six top ranked RF were concordant between readers, these included both Shape and first-order types (Table 1). Among the first-order RF, we found redundancy between Median and Mean: Median was selected because it showed higher ranking and ICC. The model with 5 RF was synthetized into a unique continuous scale that expressed values of RSign (range − 5.24–3.69). Median of RSign was different between readers, while maintaining quite similar IQR and very good ICC 0.79 (Table 1).

Univariate stratification of risk by continuous range of RSign (1-unit increment) performed slightly different between R1 (HR 1.37 95%CI 1.15–1.62, *p* < 0.001) and R2 (HR 1.28 95%CI 1.09–1.50, *p* = 0.002), still both readers maintained statistical significance. Univariate Cox Regression selected the same significant variables for both readers, namely: RSign, satellite lesions, and distant metastases. RSign showed AUC 0.73 for R1 and AUC 0.66 for R2, the optimal cut-off value for dichotomization of risk categories was:

- RSign* = 0.39 for R1.
- RSign* = 0.57 for R2.

**Fig. 2:**
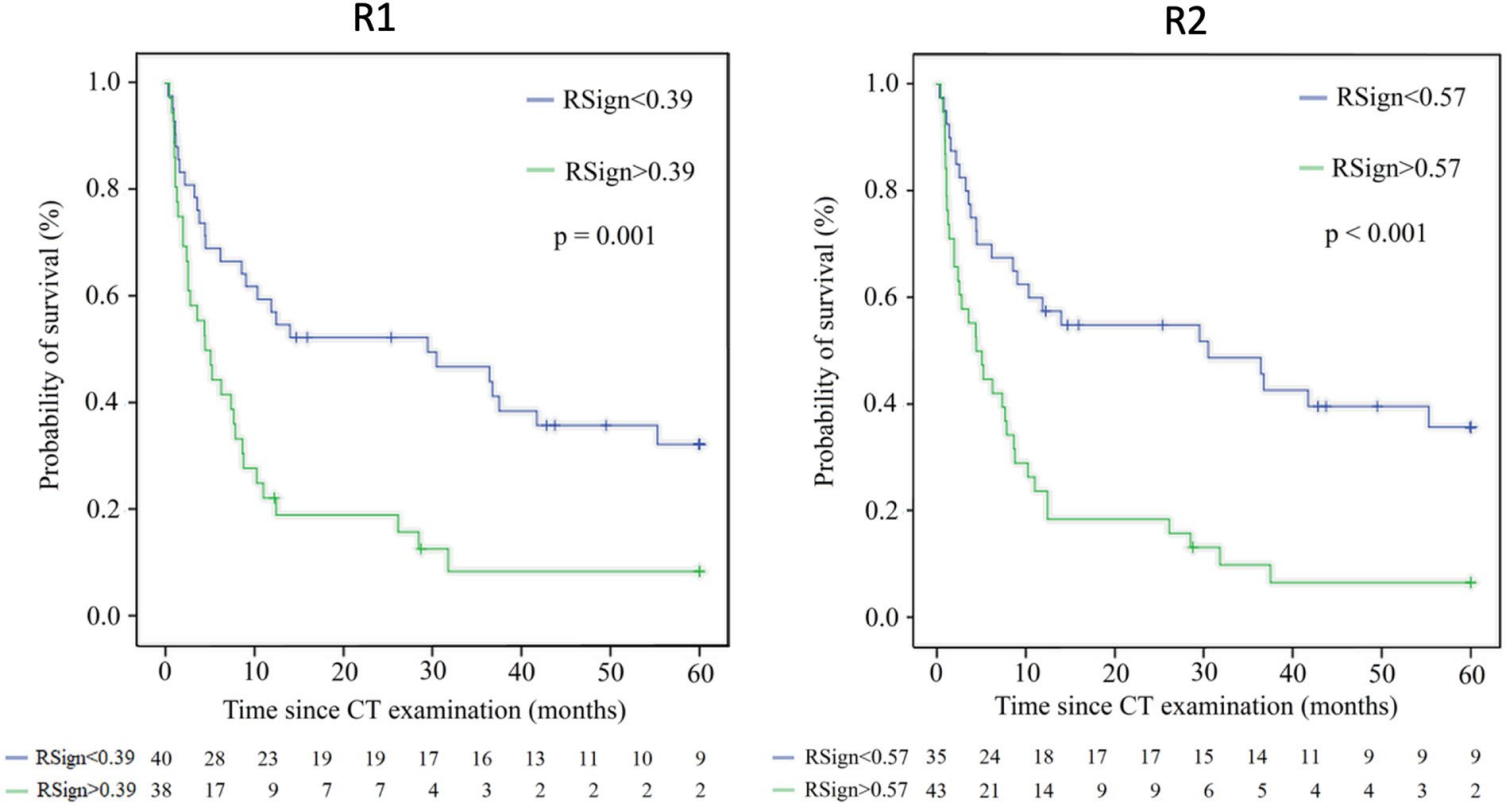
Kaplan–Meier plots estimate overall survival for low and high-risk groups, based on RSign* for each reader

According to RSign* reference, high-risk patients were distributed as follows: R1 38/78 (49%) and R2 43/78 (55%) patients. Median OS of risk categories by RSign* was significantly shorter in high-risk patients than low-risk patients (145 days vs 465 days, *p* < 0.001) and Kaplan–Meier survival curves showed significantly different risk strata for both readers (Fig. 2).

#### Risk models

Multivariate Cox proportional hazards regression analyses based on RF-R1 or RF-R2 showed different significant variables, except for RSign* (Table 2). Notably, RSign* was retained for both readers (*p* ≤ 0.001), with the highest HR among significant predictors of outcome (R1 HR 1.53 95%CI 1.24–1.88, R2 HR 1.28 95%CI 1.07–1.52—Table 2). Risk models were composed as follows:

- Model 1: satellite lesion and distant metastasis.
- Model 2: satellite lesion, distant metastasis, and RSign*.

**Table 2:** Multivariate Cox proportional regression in the whole patient cohort, and selected variables for Model 2

	R1		R2	
	Hazard ratio (95% CI)	*p* value	Hazard ratio (95% CI)	*p* value
*All predictors*				
RSign	1.53 (1.24–1.88)	** < 0.001**	1.28 (1.07–1.52)	**0.006**
Age	1.04 (1.01–1.07)	**0.02**	1.03 (1.00–1.06)	**0.05**
Gender (male)	1.58 (0.84–2.98)	0.16	1.05 (0.59–1.89)	0.86
Grading	1.42 (0.83–2.44)	0.20	1.38 (0.81–2.34)	0.24
Satellite lesions	1.70 (0.91–3.16)	0.09	2.36 (1.32–4.23)	**0.004**
Metastatic lymph nodes	1.22 (0.66–2.25)	0.54	1.23 (0.66–2.27)	0.51
Distant metastasis	2.02 (1.07–3.84)	**0.03**	1.46 (0.78–2.74)	0.23
Significant predictors*				
RSign	1.36 (1.13–1.62)	< 0.001	1.24 (1.04–1.47)	0.02
Satellite lesions	2.10 (1.17–3.82)	0.01	2.53 (1.43–4.47)	0.001
Distant metastasis	1.76 (0.97–3.20)	0.06	1.34 (0.76–2.38)	0.31

Bold is used when a statistically significant *p* value is encountered (< 0.05)

*Selected by univariate Cox regression

**Fig. 3:**
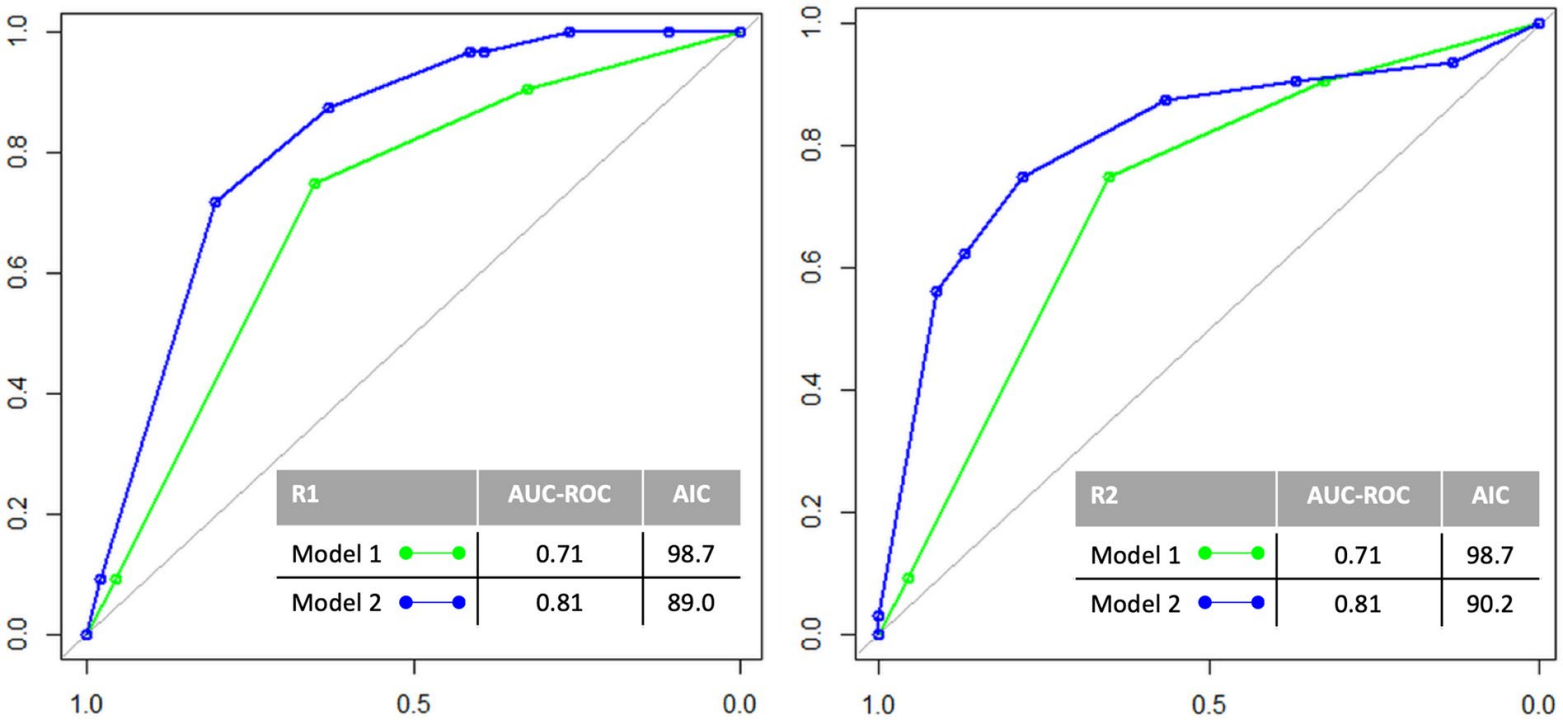
ROC curves of Model 1 and Model 2 with respective AUC and AIC, for each reader. Model 1 included satellite lesion and distant metastasis. Model 2 included satellite lesion, distant metastasis, and RSign*

**Fig. 4:**
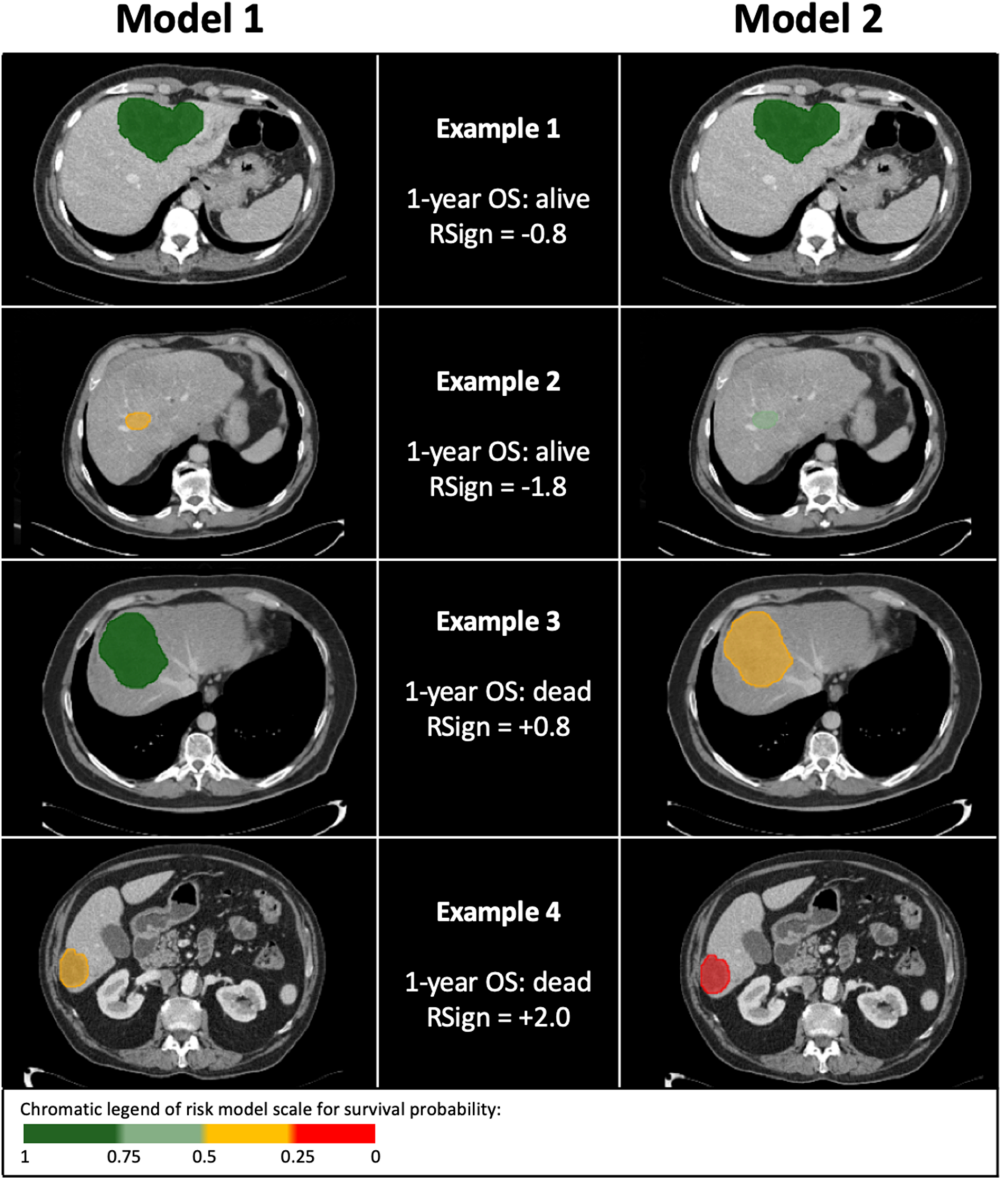
Chromatic representation of four examples with variable classification by either Model 1 or Model 2. Survival probability is reported in left column for Model 1 and right column for Model 2: the survival probability of each model is rendered by chromatic scale of the on tumor segmentation ROI (see chromatic legend in bottom box of the figure). The middle column details the outcome (alive or dead) of each case at 1 year since CT and the RSign value by reader 1. Example 1—large IMCC consistently classified with likelihood of 1-year survival > 0.75 by both Model 1 and Model 2 (RSign < 0.39), alive at 1 year. Example 2—small IMCC classified with likelihood of survival < 0.5 by Model 1 and likelihood of survival > 0.5 by Model 2 (RSign < 0.39), alive at 1 year. Example 3—large IMCC classified with high likelihood of survival > 0.75 by Model 1 and likelihood of survival < 0.5 by Model 2 (RSign > 0.39), dead at 1 year. Example 4—small IMCC classified with mid-low likelihood of survival < 0.5 by Model 1 and likelihood of survival < 0.25 by Model 2 (RSign > 0.39), dead at 1 year. Of note, example 2 and example 3 showed inconsistent risk stratification between Model 1 and Model 2. In these two cases, the inclusion of RSign (Model 2) improved the stratification of 1-year survival

Model 1 showed AUC 0.71 (both readers) for classification of 1-year survival, which improved in Model 2 (R1: AUC 0.81; R2: AUC 0.81) (Fig. 3), thus suggesting an independent prognostic yield for RSign. AIC showed relative convenience of Model 2 (Fig. 3), thus suggesting that inclusion of RSign* is worth despite increasing model complexity. Likelihood Ratio Test for Logistics models comparison was statistically significant in favor of RSign* inclusion (R1: *p* value < 0.001, R2 *p* value = 0.001). A graphic example of added value between Model 1 and Model 2 is rendered in Fig. 4.

### Analysis restricted to resected subpopulation

**Fig. 5:**
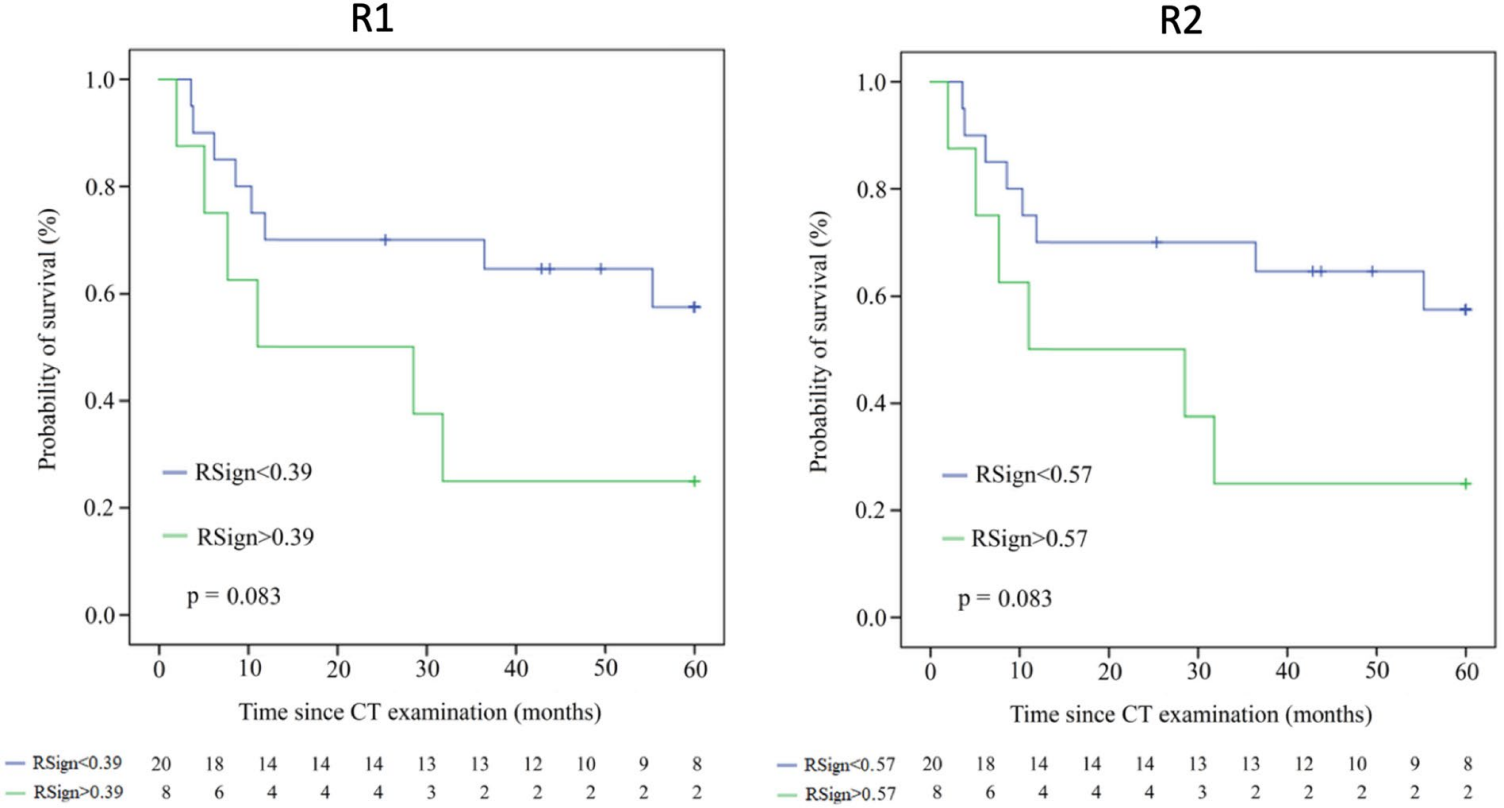
Kaplan–Meier plots estimate overall survival for low and high-risk groups, based on RSign* for each reader, in the selected population of resected IMCC

Twenty-eight patients underwent resection, they were not treated with chemotherapy. 17/28 (61%) patients died, notably 10/28 (36%) within 1 year since CT. Using RSign* derived from the overall population, median OS was significantly shorter in high-risk (601 days) than low-risk patients (1419 days, *p* ≤ 0.001), yet Kaplan–Meier survival curves were not significantly different (Fig. 5).

In this selected small population, no predictors were retained by univariate analysis. Multivariate Cox proportional hazards regression analysis selected gender and RSign, only for R1 (Table 3). Again, RSign outstood for stratification of OS (R1 1.81 95%CI 1.10–2.99, *p* = 0.02; R2 HR 1.22 95%CI 0.91–1.63, *p* = 0.19), despite widened confidence interval, as an expected statistical consequence of the population shrinkage.

**Table 3:** Multivariate Cox proportional regression for the subpopulation of patients treated by surgery

	R1		R2	
	Hazard Ratio (95% CI)	*p* value	Hazard Ratio (95% CI)	*p* value
*All predictors*				
Rsign	1.81 (1.10–2.99)	**0.02**	1.22 (0.91–1.63)	0.19
Age	1.07 (0.98–1.16)	0.12	1.06 (0.98–1.14)	0.15
Gender (male)	6.10 (1.29–28.98)	**0.02**	1.89 (0.60–5.89)	0.28
Grading	0.91 (0.29–2.87)	0.87	1.10 (0.33–3.64)	0.87
Satellite lesions	0.86 (0.24–3.10)	0.82	1.55 (0.46–5.26)	0.48
Metastatic lymph nodes	0.50 (0.15–1.64)	0.26	0.59 (0.18–1.87)	0.37
Distant metastasis	0.00 (0.00–Inf)	0.998	0.00 (0.00–Inf)	0.999

Bold is used when a statistically significant *p* value is encountered (< 0.05)

## Discussion

In this study, we showed that volume CT radiomics is associated with prognosis in patients with IMCC and that RF can be synthetized into RSign for stratification of survival by a unique scale. We stipulated a threshold for definition of high and low-risk patients by RSign, called RSign*. The risk model including RSign* outperformed the model without radiomics. Moreover, the stratification by RSign* showed a trend for differentiation of survival also in the subgroup of patients undergoing surgery.

### Variability and selection of RF

The outcome of cholangiocarcinoma is poor, its optimal clinical management is challenged by the gaze between curative approach and minimized invasive procedures. In 2017, Raoof proposed a risk model based on post-operative variables for prediction of survival in resected intrahepatic cholangiocarcinoma (MEGNA score) [30]. While showing the accuracy of MEGNA score compared to AJCC staging system, the authors underscored the need for pre-operative tools to inform decision regarding surgery and adjuvant therapy. The pre-operative characterization of intrahepatic cholangiocarcinoma burgeoned thanks to the investigation of prognostic factors from imaging, including radiomics.

In 2019, Ji proposed a nomogram by 8 RF plus CA19.9 for prediction of prognosis and, notably, for stratification of lymph node metastases beyond morphologic standards [15]. This study included a broad population with full range of treatment options, consistent with ours. In 2020, Chu reported that RF could stratify poor outcome after surgery [23]. Both Ji and Chu reported excellent interobserver agreement, yet they both analyzed only experienced readers. In particular, Chu reported excellent agreement (98.7% RF with ICC > 0.5) [23], which is substantially different from our results showing higher variability between one resident and one experience abdominal radiologist (71% RF with ICC > 0.5). Such discrepancy underscores the need for experienced reader for high-skilled segmentation of tumor boundaries. Of note, segmentation of focal abnormalities in liver is more challenging compared to other organs where semi-automatic segmentation is already used in CT clinical practice and demonstrated with good diagnostic performance also among technologists (e.g., lung nodules) [31–33]. However, despite high interobserver variability in one third of RF, we observed that the six top ranked RF were consistent between readers, and were selected for building a radiomic model with minimized interaction from manual segmentation. Of note, the selected RF could be interpreted into morphological impressions. For instance, feature “Surface volume ratio” is representative of both size and shape: this RF is expected to vary depending on lesion size (the larger the lesion the lower the value) and pattern of growth (the more irregular the surface the higher the value). Nonetheless, “Surface volume ratio” relies on manual segmentation with as low as ICC 0.54 and therefore underscores the relevance of standardized segmentation. The six selected RF were similar in kind (first-order RF including percentiles of density) to those from a previous study focusing on prediction of surgical utility by RF in portal venous phase CT [23]. This overlapping character (portal venous phase and first-order radiomics) suggests that relatively simple RF should be deemed relevant also in an unselected population like ours (including both surgery candidates and advanced disease). In line with this observation, previous studies reported association of density metrics with mutations or protein expression in intrahepatic cholangiocarcinoma [34, 35].

Morphologic variables and RF were derived from venous phase in our retrospective database, whereas previous studies selected arterial phase. Mosconi et al. reported that textural RF are best extracted from arterial phase, in a population selected for transarterial radioembolization [24]. In that study, venous phase remained significant for first-order features, including “Mean”, partly consistent with our observation. It is worth mentioning that some difference between our results and Mosconi’s might ought to different clinical characteristics that potentially influence the imaging appearance of contrast pharmacodynamics, as well as interobserver variability and technical details (contrast agent was different between our study and Mosconi’s, 400 and 350 mg I/mL, respectively). Nonetheless, comparison of these studies shows that first-order features warrant risk stratification, and this was consistent across our readers. First of its kind, our experiment also detailed the variability of radiomics across CT scanners and CT protocols, which led to selection of the most robust RF: this selection warrants stability of RSign for clinical use.

### Radiomic signature

We propose a simple RSign with 5 RF into a synthetized single score, which is optimal for practical use and similar to the approach formerly proposed for radiomics of primary liver malignancies [15, 36, 37]. The univariate stratification of survival by RSign granted similar magnitude of risk in each reader of this study, about 1.4-fold for R1 and 1.3-fold for R2 (Table 2). This was true when analyzing RSign with “relative approach”, with nominal increment of 1-unit throughout the full range of RSign. However, “relative approach” has limited value for clinical translation, because clinical decision making is best served by discrete categories defined by absolute threshold. The analysis of RSign with absolute approach showed interobserver variability in this study: RSign* was different between readers. The interpretation of such difference is allegedly found in the aforementioned variable experience. To the best of our knowledge, the individual performance by discrete categories of risk was not reported in the literature. Our data fill in this gap by showing the variability of absolute threshold when RF are used by readers with different experience. The translation of absolute threshold into practical use depends on optimized segmentation method. Zhao showed that interobserver variability is mitigated by semi-automated segmentation of the neoplastic volume [38]. Eventually, semi-automated tools will cope with segmentation bias and reconcile variability of risk strata within clinically applicable boundaries.

We used a multistep statistical process to integrate RSign* in risk models including standard clinical variables. Risk models with RSign* performed better than model without RSign*, with statistically significant improvement for both readers. Inclusion of RSign* was confirmed statistically worth by AIC analysis. Of note, standard clinical variables retained in model were represented by morphological descriptors on CT. Also previous studies retained findings from CT morphologic domain (e.g., lymph node metastases, liver metastases) [15, 23, 24], and discarded demographic and laboratory data. This consistency among independent studies brings about the most prominent role of CT imaging for stratification of disease course.

### RSign in candidates for surgery

We analyzed our method in a subpopulation of patients who underwent resection, with the aim of testing our general approach into specific risk strata of IMCC. The selection of this subpopulation was driven by previous evidence showing that operability is an independent prognostic factor [39]. The yield of RSign by 5 RF derived from the whole population of our study was confirmed in selected subpopulation undergoing surgery. Furthermore, RSign* could classify patients with fairly different survival among those undergoing surgical resection. This observation shows that RSign is not dependent on current standards for disease management and prognostication, and allegedly it projects RSign for complementing morphological characterization.

### Limitations

The current study suffers from limitations. First, a single phase of contrast enhancement was explored. Second, we could not perform external validation. Third, the small absolute number of patients represented power limitation, hence we cannot exclude type II errors on second or higher order RF. Finally, the small population of this study could be investigated with only one reference RSign*, resulting in binary strata. However, the optimal clinical support is expected from tools multiple discrete strata, including indeterminate category. Larger studies are warranted to investigate polychromatic interpretation of RSign.

In conclusion, we proposed a RSign that associated with survival in IMCC. The proposed RSign was discretized in radiomic categories of risk and its yield complemented in multivariable risk models. The reported interobserver variability of RSign* warns on the need for consistent segmentation of IMCC on CT images. The generalized derivation of RSign* showed potential for use also in subpopulation undergoing surgery.

## Supplementary Information

Below is the link to the electronic supplementary material.

Supplementary file1 (DOCX 104 KB)

## Abbreviations

AIC: Akaike information criterion
AJCC/UICC: American Joint Committee on Cancer/Union for International Cancer Control
AUC: Area under the curve
CT: Compute tomography
GLCM: Gray-Level-Co-occurrence-Matrix
GLDM: Gray-Level-Dependence-Matrix
GLRLM: Gray-Level-Run-Length-Matrix
GLSZM: Gray-Level-Size-Zone-Matrix
ICC: Intraclass correlation coefficient
IMCC: Intrahepatic mass-forming cholangiocarcinoma
IQR: Interquartile range
NGTDM: Neighboring-Gray-Tone-Difference-Matrix
OS: Overall survival
PCA: Principal component analysis
RF: Radiomic features
ROC: Receiver Operating Characteristic
RSign: Radiomic signature
VOI: Volume of interest
