# Supplementary material for: Integrated prognostication of intrahepatic cholangiocarcinoma by contrast-enhanced computed tomography: the adjunct yield of radiomics
Source: Abdom Radiol (NY). 2021 Jun 24;46(10):4689–700. doi: 10.1007/s00261-021-03183-9 (PMC8435517; doi:10.1007/s00261-021-03183-9)
Supplement: Supplementary file 1 — Supplementary file1 (DOCX 104 KB) [file 261_2021_3183_MOESM1_ESM.docx]

Supplementary material

### Analysis restricted to resected subpopulation

The RSign was tested in the subpopulation of patients undergoing surgery (without chemotherapy), with the aim of describing the prognostic yield of the generalized RSign in a distilled homogeneous population with relatively good prognosis and for which long-term prediction of survival by radiomics might be most relevant. The above analysis was iterated on this selection and the two risk subgroups were obtained by using the *RSign** value formerly determined for the whole cohort analysis.

## Supplementary Table 1

Details of interobserver variability on CT imaging variables from clinical standard.

| **Variable** | **Frequency of discrepancy** | **Consensus frequency in favor of R1** | **R1** | | **R2** | |
| --- | --- | --- | --- | --- | --- | --- |
|  |  |  | **FN** | **FP** | **FN** | **FP** |
| Satellite hepatic lesions | 7.8% (6/78) | 83.3% (5/6) | 1 | 0 | 3 | 2 |
| Lymph node metastasis | 10.3% (8/78) | 87.5% (7/8) | 1 | 0 | 4 | 3 |
| Distant metastasis | 6.4% (5/78) | 60% (2/5) | 2 | 0 | 2 | 1 |
| Maximum diameter on axial plane | 7.8% (6/78) | N.A. | N.A. | N.A. | N.A. | N.A. |

*Legend: FN, false negative; FP, false positive.*

## Supplementary Table 2

Details of CT protocols (acquisition and reconstruction) along with the selected test for analysis of radiomic features (RF) variability.

| **Parameter** | **Result** | | **Statistical test** |
| --- | --- | --- | --- |
| Scanner model | Values | SIEMENS Emotion 6 | Kruskal-Wallis |
|  |  | SIEMENS Sensation 64 |  |
|  |  | SIEMENS Definition Flash |  |
| Tube voltage | Range | 100-140 | Kruskal-Wallis |
| Pitch | Range | 0,6-1,3 | Spearman |
| Pixel Spacing | Range | 0,59-0,96 | Spearman |
| Slice thickness | Range | 1,5-2,5 | Kruskal-Wallis |
| Reconstruction algorithm | Values | B20f  B25f  B30f  B30s^a^  B31s  B40s ^a^ | Kruskal-Wallis |

^a^ Excluded from statistical analysis because of minimum representation.

## Supplementary Table 3

Variability of RF according to CT protocol details.

| Type | Name | Scanner model | Reconstruction algorithm | Tube voltage | Slice thickness | Pitch | Pixel Spacing |
| --- | --- | --- | --- | --- | --- | --- | --- |
|  |  | *P* | *P* | *P* | *P* | *P* | *P* |
| Mask-original | VoxelNum | 0.58 | 0.21 | 0.65 | 0.46 | 0.33 | 0.88 |
| shape | VoxelVolume | 0.40 | 0.08 | 0.65 | 0.46 | 0.26 | 0.61 |
| shape | Maximum3DDiameter | 0.21 | 0.21 | 0.65 | 0.46 | 0.18 | 0.54 |
| shape | MeshVolume | 0.40 | 0.08 | 0.65 | 0.46 | 0.26 | 0.61 |
| shape | MajorAxisLength | 0.40 | 0.08 | 0.65 | 0.71 | 0.11 | 0.36 |
| shape | Sphericity | 0.86 | 0.02 | 0.83 | 0.76 | 0.65 | 0.46 |
| shape | LeastAxisLength | 0.58 | 0.08 | 0.65 | 0.46 | 0.32 | 0.68 |
| shape | Elongation | 0.58 | 0.21 | 0.83 | 0.98 | 0.11 | 0.60 |
| shape | SurfaceVolumeRatio | 0.86 | 0.25 | 0.83 | 0.76 | 0.26 | 0.75 |
| shape | Maximum2DDiameterSlice | 0.09 | 0.08 | 0.60 | 0.71 | 0.32 | 0.43 |
| shape | Flatness | 0.14 | 0.60 | 0.27 | 0.18 | 0.39 | 0.13 |
| shape | SurfaceArea | 0.21 | 0.08 | 0.65 | 0.46 | 0.29 | 0.68 |
| shape | MinorAxisLength | 0.40 | 0.02 | 0.65 | 0.71 | 0.28 | 0.50 |
| shape | Maximum2DDiameterColumn | 0.21 | 0.08 | 0.65 | 0.46 | 0.15 | 0.47 |
| shape | Maximum2DDiameterRow | 0.58 | 0.08 | 0.65 | 0.46 | 0.25 | 0.59 |
| gldm | GrayLevelVariance | 0.66 | 0.21 | 0.71 | 0.22 | 0.81 | 0.50 |
| gldm | HighGrayLevelEmphasis | 0.54 | 0.45 | 0.64 | 0.45 | 0.09 | 0.74 |
| gldm | DependenceEntropy | 0.05 | 0.25 | 0.29 | 0.46 | 0.34 | 0.54 |
| gldm | DependenceNonUniformity | 0.40 | 0.21 | 0.65 | 0.46 | 0.41 | 0.85 |
| gldm | GrayLevelNonUniformity | 0.40 | 0.21 | 0.65 | 0.71 | 0.29 | 0.83 |
| gldm | SmallDependenceEmphasis | 0.08 | 0.60 | 0.15 | 0.46 | 0.31 | 0.60 |
| gldm | SmallDependenceHighGrayLevelEmphasis | 0.91 | 0.45 | 0.22 | 0.35 | 0.38 | 0.67 |
| gldm | DependenceNonUniformityNormalized | 0.70 | 0.21 | 0.71 | 0.66 | 0.12 | 0.96 |
| gldm | LargeDependenceEmphasis | 0.30 | 0.45 | 0.71 | 0.43 | 0.16 | 0.70 |
| gldm | LargeDependenceLowGrayLevelEmphasis | 0.91 | 0.06 | 0.27 | 0.20 | 0.23 | 0.85 |
| gldm | DependenceVariance | 0.58 | 0.45 | 0.71 | 0.76 | 0.12 | 0.92 |
| gldm | LargeDependenceHighGrayLevelEmphasis | 0.14 | 0.60 | 0.07 | 0.23 | 0.05 | 0.77 |
| gldm | SmallDependenceLowGrayLevelEmphasis | 0.10 | 0.60 | 0.07 | 0.13 | 0.10 | 0.51 |
| gldm | LowGrayLevelEmphasis | 0.38 | 0.45 | 0.64 | 0.43 | 0.07 | 0.71 |
| glcm | JointAverage | 0.54 | 0.45 | 0.64 | 0.45 | 0.08 | 0.69 |
| glcm | SumAverage | 0.54 | 0.45 | 0.64 | 0.45 | 0.08 | 0.69 |
| glcm | JointEntropy | 0.86 | 0.21 | 0.81 | 0.66 | 0.65 | 0.50 |
| glcm | ClusterShade | 0.71 | 0.60 | 0.69 | 0.94 | 0.05 | 0.48 |
| glcm | MaximumProbability | 0.54 | 0.45 | 0.71 | 0.81 | 0.61 | 0.42 |
| glcm | Idmn | 0.54 | 0.49 | 0.08 | 0.81 | 0.29 | 0.35 |
| glcm | JointEnergy | 0.91 | 0.21 | 0.81 | 0.43 | 0.65 | 0.34 |
| glcm | Contrast | 0.91 | 0.45 | 0.71 | 0.94 | 0.33 | 0.71 |
| glcm | DifferenceEntropy | 0.86 | 0.60 | 0.83 | 0.85 | 0.35 | 0.68 |
| glcm | InverseVariance | 0.86 | 0.45 | 0.83 | 0.66 | 0.14 | 0.86 |
| glcm | DifferenceVariance | 0.86 | 0.60 | 0.81 | 0.85 | 0.33 | 0.64 |
| glcm | Idn | 0.54 | 0.45 | 0.08 | 0.85 | 0.29 | 0.42 |
| glcm | Idm | 0.97 | 0.45 | 0.71 | 0.98 | 0.30 | 0.73 |
| glcm | Correlation | 0.12 | 0.49 | 0.21 | 0.53 | 0.77 | 0.75 |
| glcm | Autocorrelation | 0.54 | 0.45 | 0.64 | 0.45 | 0.09 | 0.69 |
| glcm | SumEntropy | 0.58 | 0.25 | 0.71 | 0.71 | 0.97 | 0.65 |
| glcm | MCC | 0.54 | 0.25 | 0.69 | 0.85 | 0.97 | 0.91 |
| glcm | SumSquares | 0.66 | 0.25 | 0.71 | 0.22 | 0.74 | 0.59 |
| glcm | ClusterProminence | 0.40 | 0.45 | 0.65 | 0.71 | 0.78 | 0.72 |
| glcm | Imc2 | 0.15 | 0.49 | 0.31 | 0.81 | 0.76 | 0.60 |
| glcm | Imc1 | 0.19 | 0.49 | 0.31 | 0.94 | 0.62 | 0.70 |
| glcm | DifferenceAverage | 0.91 | 0.45 | 0.71 | 0.94 | 0.34 | 0.74 |
| glcm | Id | 0.97 | 0.45 | 0.71 | 0.98 | 0.28 | 0.75 |
| glcm | ClusterTendency | 0.58 | 0.49 | 0.69 | 0.71 | 0.82 | 0.67 |
| firstorder | InterquartileRange | 0.11 | 0.49 | 0.37 | 0.30 | 0.96 | 0.32 |
| firstorder | Skewness | 0.66 | 0.60 | 0.34 | 0.71 | 0.10 | 0.45 |
| firstorder | Uniformity | 0.70 | 0.21 | 0.71 | 0.30 | 0.83 | 0.56 |
| firstorder | Median | 1.00 | 0.54 | 0.72 | 0.61 | 0.41 | 0.87 |
| firstorder | Energy | 0.71 | 0.21 | 0.83 | 0.94 | 0.28 | 0.83 |
| firstorder | RobustMeanAbsoluteDeviation | 0.66 | 0.49 | 0.71 | 0.71 | 0.90 | 0.37 |
| firstorder | MeanAbsoluteDeviation | 0.66 | 0.25 | 0.71 | 0.46 | 0.82 | 0.47 |
| firstorder | TotalEnergy | 0.42 | 0.25 | 0.83 | 0.94 | 0.19 | 0.56 |
| firstorder | Maximum | 0.75 | 0.62 | 0.29 | 0.90 | 0.32 | 0.12 |
| firstorder | RootMeanSquared | 0.97 | 0.45 | 0.71 | 0.76 | 0.63 | 0.81 |
| firstorder | 90Percentile | 0.79 | 0.21 | 0.69 | 0.40 | 0.79 | 0.93 |
| firstorder | Minimum | 0.32 | 0.60 | 0.43 | 0.18 | 0.08 | 0.79 |
| firstorder | Entropy | 0.66 | 0.21 | 0.71 | 0.22 | 0.78 | 0.52 |
| firstorder | Range | 0.43 | 0.51 | 0.20 | 0.27 | 0.44 | 0.55 |
| firstorder | Variance | 0.66 | 0.21 | 0.71 | 0.22 | 0.85 | 0.48 |
| firstorder | 10Percentile | 0.97 | 0.21 | 0.71 | 0.18 | 0.39 | 0.60 |
| firstorder | Kurtosis | 0.38 | 0.60 | 0.10 | 0.53 | 0.30 | 0.68 |
| firstorder | Mean | 0.86 | 0.45 | 0.71 | 0.76 | 0.48 | 0.79 |
| glrlm | ShortRunLowGrayLevelEmphasis | 0.31 | 0.60 | 0.29 | 0.18 | 0.07 | 0.61 |
| glrlm | GrayLevelVariance | 0.58 | 0.21 | 0.71 | 0.46 | 0.81 | 0.51 |
| glrlm | LowGrayLevelRunEmphasis | 0.38 | 0.45 | 0.64 | 0.43 | 0.08 | 0.69 |
| glrlm | GrayLevelNonUniformityNormalized | 0.70 | 0.21 | 0.71 | 0.30 | 0.84 | 0.63 |
| glrlm | RunVariance | 0.30 | 0.45 | 0.71 | 0.43 | 0.14 | 0.83 |
| glrlm | GrayLevelNonUniformity | 0.40 | 0.21 | 0.65 | 0.46 | 0.38 | 0.86 |
| glrlm | LongRunEmphasis | 0.30 | 0.45 | 0.71 | 0.43 | 0.14 | 0.77 |
| glrlm | ShortRunHighGrayLevelEmphasis | 0.54 | 0.21 | 0.64 | 0.45 | 0.11 | 0.84 |
| glrlm | RunLengthNonUniformity | 0.40 | 0.06 | 0.65 | 0.46 | 0.39 | 0.91 |
| glrlm | ShortRunEmphasis | 0.19 | 0.60 | 0.31 | 0.66 | 0.20 | 0.63 |
| glrlm | LongRunHighGrayLevelEmphasis | 0.38 | 0.60 | 0.29 | 0.23 | 0.04 | 0.67 |
| glrlm | RunPercentage | 0.42 | 0.45 | 0.71 | 0.66 | 0.15 | 0.70 |
| glrlm | LongRunLowGrayLevelEmphasis | 0.91 | 0.21 | 0.27 | 0.43 | 0.18 | 0.90 |
| glrlm | RunEntropy | 0.06 | 0.25 | 0.10 | 0.07 | 0.41 | 0.89 |
| glrlm | HighGrayLevelRunEmphasis | 0.54 | 0.45 | 0.64 | 0.45 | 0.10 | 0.73 |
| glrlm | RunLengthNonUniformityNormalized | 0.42 | 0.60 | 0.31 | 0.66 | 0.18 | 0.62 |
| glszm | GrayLevelVariance | 0.70 | 0.21 | 0.60 | 0.13 | 0.80 | 0.50 |
| glszm | ZoneVariance | 0.71 | 0.25 | 0.55 | 0.81 | 0.25 | 0.84 |
| glszm | GrayLevelNonUniformityNormalized | 0.15 | 0.21 | 0.67 | 0.85 | 0.31 | 0.71 |
| glszm | SizeZoneNonUniformityNormalized | 0.31 | 0.45 | 0.34 | 0.37 | 0.48 | 0.85 |
| glszm | SizeZoneNonUniformity | 0.25 | 0.01 | 0.34 | 0.22 | 0.39 | 0.98 |
| glszm | GrayLevelNonUniformity | 0.58 | 0.06 | 0.65 | 0.22 | 0.43 | 0.84 |
| glszm | LargeAreaEmphasis | 0.71 | 0.25 | 0.55 | 0.81 | 0.24 | 0.84 |
| glszm | SmallAreaHighGrayLevelEmphasis | 0.54 | 0.21 | 0.27 | 0.53 | 0.18 | 0.78 |
| glszm | ZonePercentage | 0.07 | 0.49 | 0.71 | 0.66 | 0.28 | 0.65 |
| glszm | LargeAreaLowGrayLevelEmphasis | 0.97 | 0.25 | 0.71 | 0.76 | 0.70 | 0.64 |
| glszm | LargeAreaHighGrayLevelEmphasis | 0.71 | 0.25 | 0.83 | 0.66 | 0.09 | 0.93 |
| glszm | HighGrayLevelZoneEmphasis | 0.54 | 0.21 | 0.23 | 0.53 | 0.21 | 0.65 |
| glszm | SmallAreaEmphasis | 0.31 | 0.45 | 0.34 | 0.37 | 0.49 | 0.84 |
| glszm | LowGrayLevelZoneEmphasis | 0.66 | 0.60 | 0.69 | 0.46 | 0.13 | 0.54 |
| glszm | ZoneEntropy | 0.58 | 0.06 | 0.83 | 0.38 | 0.82 | 0.51 |
| glszm | SmallAreaLowGrayLevelEmphasis | 0.58 | 0.60 | 0.69 | 0.43 | 0.19 | 0.50 |
| ngtdm | Coarseness | 0.54 | 0.21 | 0.65 | 0.57 | 0.43 | 0.83 |
| ngtdm | Complexity | 0.70 | 0.06 | 0.27 | 0.28 | 0.48 | 0.65 |
| ngtdm | Strength | 0.86 | 0.25 | 0.81 | 0.94 | 0.45 | 0.84 |
| ngtdm | Contrast | 0.06 | 0.60 | 0.10 | 0.46 | 0.20 | 0.37 |
| ngtdm | Busyness | 0.91 | 0.25 | 0.81 | 0.98 | 0.52 | 0.70 |

## Supplementary Table 4

Inter-observer variability of RF. Feature selection was operated with ICC>0.5.

ICC chromatic scale

0 0.25 0.50 0.75 1

| Type | Name | R1 | | | R2 | | | ICC |
| --- | --- | --- | --- | --- | --- | --- | --- | --- |
|  |  | *Median* | *1st Quartile* | *3rd Quartile* | *Median* | *1st Quartile* | *3rd Quartile* |  |
| Mask-original | VoxelNum | 95189 | 21363 | 253514 | 89907 | 28165 | 259946.25 | 0.66 |
| shape | VoxelVolume | 83341.34 | 24753.61 | 273302.25 | 75101.68 | 31390.22 | 256197.59 | 0.686 |
| shape | Maximum3DDiameter | 86.74 | 55.03 | 127.47 | 83.33 | 60.86 | 124.27 | 0.663 |
| shape | MeshVolume | 83113.83 | 24036.8 | 274490.5 | 75000.38 | 31300.15 | 256044.34 | 0.686 |
| shape | MajorAxisLength | 66.23 | 44.11 | 101.31 | 67.59 | 46.46 | 96.3 | 0.703 |
| shape | Sphericity | 0.57 | 0.49 | 0.67 | 0.6 | 0.55 | 0.65 | 0.095 |
| shape | LeastAxisLength | 40.21 | 25.18 | 53.05 | 40 | 25.47 | 55.34 | 0.752 |
| shape | Elongation | 0.79 | 0.7 | 0.86 | 0.79 | 0.73 | 0.87 | 0.443 |
| shape | SurfaceVolumeRatio | 0.2 | 0.14 | 0.29 | 0.19 | 0.13 | 0.29 | 0.54 |
| shape | Maximum2DDiameterSlice | 74.4 | 48.67 | 114.68 | 71.79 | 52.76 | 110.58 | 0.664 |
| shape | Flatness | 0.59 | 0.48 | 0.68 | 0.6 | 0.53 | 0.67 | 0.457 |
| shape | SurfaceArea | 19029.81 | 6485.73 | 36978.1 | 14003.78 | 8264.04 | 31824 | 0.682 |
| shape | MinorAxisLength | 48.91 | 33.77 | 75.94 | 48.02 | 37.65 | 74.82 | 0.734 |
| shape | Maximum2DDiameterColumn | 78.39 | 48.71 | 103.9 | 71.92 | 52.59 | 105.66 | 0.681 |
| shape | Maximum2DDiameterRow | 72.23 | 48.06 | 104.27 | 68.18 | 52.94 | 101.61 | 0.759 |
| firstorder | InterquartileRange | 40 | 33 | 43 | 40 | 36 | 45 | 0.161 |
| firstorder | Skewness | -0.06 | -0.26 | 0.06 | -0.5 | -1.68 | -0.04 | 0.249 |
| firstorder | Uniformity | 0.24 | 0.22 | 0.27 | 0.23 | 0.21 | 0.25 | 0.645 |
| firstorder | Median | 70 | 59 | 89 | 70 | 60 | 89.75 | 0.669 |
| firstorder | Energy | 534498300 | 201267984 | 1394840532 | 590105197 | 255403964 | 1548040107 | 0.651 |
| firstorder | RobustMeanAbsoluteDeviation | 16.46 | 14 | 17.92 | 17.05 | 15.23 | 19.48 | 0.857 |
| firstorder | MeanAbsoluteDeviation | 22.99 | 20.21 | 25.04 | 25.3 | 22.56 | 28.51 | 0.339 |
| firstorder | TotalEnergy | 431260102 | 206044028 | 1517948911 | 464560617 | 227033174 | 1660238301 | 0.886 |
| firstorder | Maximum | 194 | 176 | 223 | 211.5 | 184.25 | 259 | 0.829 |
| firstorder | RootMeanSquared | 76.5 | 65.56 | 90.46 | 76.56 | 69.4 | 92.3 | 0.228 |
| firstorder | 90Percentile | 107 | 94 | 120 | 110 | 97.25 | 125.25 | 0.862 |
| firstorder | Minimum | -90 | -140 | -50 | -145 | -659 | -93.75 | 0.222 |
| firstorder | Entropy | 2.28 | 2.12 | 2.41 | 2.37 | 2.24 | 2.54 | 0.534 |
| firstorder | Range | 279 | 233 | 360 | 367.5 | 290.5 | 1029.5 | 0.285 |
| firstorder | Variance | 836.11 | 673.66 | 1023.07 | 1181.91 | 842.98 | 1524.81 | 0.259 |
| firstorder | 10Percentile | 36 | 21 | 47 | 34 | 19.25 | 45.25 | 0.259 |
| firstorder | Kurtosis | 3.25 | 3 | 4.08 | 6.14 | 3.26 | 35.17 | 0.733 |
| firstorder | Mean | 70.55 | 59.02 | 85.86 | 69.07 | 59 | 87.54 | 0.037 |
| secondorder | GrayLevelVariance | 1.41 | 1.16 | 1.72 | 1.98 | 1.44 | 2.52 | 0.766 |
| secondorder | HighGrayLevelEmphasis | 55.64 | 36.86 | 85.37 | 96.28 | 59.52 | 889.23 | 0.618 |
| secondorder | DependenceEntropy | 6.14 | 5.92 | 6.29 | 6.29 | 6.08 | 6.44 | 0.769 |
| secondorder | DependenceNonUniformity | 5884.03 | 1516.96 | 14730.17 | 5682.66 | 1785.45 | 15587.64 | 0.709 |
| secondorder | GrayLevelNonUniformity | 25758.24 | 5457.71 | 67013.49 | 22647.05 | 6924.42 | 58773.59 | 0.801 |
| secondorder | SmallDependenceEmphasis | 0.04 | 0.03 | 0.05 | 0.04 | 0.03 | 0.05 | 0.882 |
| secondorder | SmallDependenceHighGrayLevelEmphasis | 2.4 | 1.69 | 4.03 | 4.48 | 2.26 | 34.34 | 0.412 |
| secondorder | DependenceNonUniformityNormalized | 0.07 | 0.06 | 0.07 | 0.07 | 0.06 | 0.07 | 0.571 |
| secondorder | LargeDependenceEmphasis | 111.6 | 85.83 | 144.48 | 110.41 | 87.71 | 146.75 | 0.836 |
| secondorder | LargeDependenceLowGrayLevelEmphasis | 2.13 | 1.27 | 3.55 | 1.11 | 0.13 | 2.24 | 0.665 |
| secondorder | DependenceVariance | 17.47 | 13.93 | 21.29 | 17.27 | 14.16 | 21.57 | 0.249 |
| secondorder | LargeDependenceHighGrayLevelEmphasis | 5821.21 | 3333.88 | 10254.46 | 10552.72 | 5716.96 | 114304.99 | 0.659 |
| secondorder | SmallDependenceLowGrayLevelEmphasis | 0 | 0 | 0 | 0 | 0 | 0 | 0.527 |
| secondorder | LowGrayLevelEmphasis | 0.02 | 0.01 | 0.03 | 0.01 | 0 | 0.02 | 0.187 |
| secondorder | JointAverage | 7.33 | 5.94 | 9.19 | 9.66 | 7.54 | 29.78 | 0.218 |
| secondorder | SumAverage | 14.67 | 11.89 | 18.38 | 19.32 | 15.08 | 59.56 | 0.762 |
| secondorder | JointEntropy | 4.23 | 3.92 | 4.55 | 4.35 | 4.14 | 4.73 | 0.767 |
| secondorder | ClusterShade | -0.19 | -2.14 | 1.18 | -5.74 | -20.75 | -0.14 | 0.848 |
| secondorder | MaximumProbability | 0.14 | 0.11 | 0.18 | 0.14 | 0.11 | 0.17 | 0.836 |
| secondorder | Idmn | 0.99 | 0.99 | 0.99 | 0.99 | 0.99 | 1 | 0.147 |
| secondorder | JointEnergy | 0.07 | 0.06 | 0.09 | 0.07 | 0.06 | 0.08 | 0.733 |
| secondorder | Contrast | 1.23 | 0.97 | 1.68 | 1.41 | 1.09 | 1.87 | 0.099 |
| secondorder | DifferenceEntropy | 1.55 | 1.42 | 1.72 | 1.6 | 1.45 | 1.75 | 0.738 |
| secondorder | InverseVariance | 0.5 | 0.49 | 0.51 | 0.5 | 0.49 | 0.5 | 0.796 |
| secondorder | DifferenceVariance | 0.56 | 0.48 | 0.73 | 0.65 | 0.53 | 0.86 | 0.437 |
| secondorder | Idn | 0.94 | 0.93 | 0.95 | 0.95 | 0.94 | 0.98 | 0.72 |
| secondorder | Idm | 0.63 | 0.59 | 0.67 | 0.62 | 0.59 | 0.67 | 0.566 |
| secondorder | Correlation | 0.51 | 0.4 | 0.61 | 0.62 | 0.5 | 0.68 | 0.49 |
| secondorder | Autocorrelation | 54.47 | 36.16 | 84.64 | 94.89 | 57.4 | 887.57 | 0.585 |
| secondorder | SumEntropy | 3.06 | 2.87 | 3.19 | 3.15 | 3.01 | 3.34 | 0.766 |
| secondorder | MCC | 0.57 | 0.44 | 0.66 | 0.73 | 0.58 | 0.8 | 0.775 |
| secondorder | SumSquares | 1.37 | 1.14 | 1.69 | 1.78 | 1.4 | 2.42 | 0.271 |
| secondorder | ClusterProminence | 59.18 | 36.7 | 86.23 | 236.59 | 69.98 | 927.22 | 0.672 |
| secondorder | Imc2 | 0.6 | 0.47 | 0.69 | 0.67 | 0.56 | 0.75 | 0.564 |
| secondorder | Imc1 | -0.11 | -0.15 | -0.07 | -0.13 | -0.18 | -0.1 | 0.16 |
| secondorder | DifferenceAverage | 0.82 | 0.71 | 0.97 | 0.85 | 0.72 | 0.97 | 0.702 |
| secondorder | Id | 0.65 | 0.62 | 0.69 | 0.65 | 0.62 | 0.69 | 0.586 |
| secondorder | ClusterTendency | 4.24 | 3.36 | 5.12 | 5.76 | 4.32 | 7.64 | 0.779 |
| secondorder | ShortRunLowGrayLevelEmphasis | 0.02 | 0.01 | 0.03 | 0.01 | 0 | 0.02 | 0.323 |
| secondorder | GrayLevelVariance | 1.6 | 1.33 | 1.92 | 2.22 | 1.59 | 3.1 | 0.135 |
| secondorder | LowGrayLevelRunEmphasis | 0.02 | 0.01 | 0.03 | 0.01 | 0 | 0.02 | 0.286 |
| secondorder | GrayLevelNonUniformityNormalized | 0.22 | 0.21 | 0.25 | 0.21 | 0.19 | 0.23 | 0.694 |
| secondorder | RunVariance | 0.87 | 0.62 | 1.26 | 0.85 | 0.64 | 1.27 | 0.842 |
| secondorder | GrayLevelNonUniformity | 16938.38 | 3561.87 | 36257.74 | 12424.48 | 4408.88 | 33210.27 | 0.674 |
| secondorder | LongRunEmphasis | 3.15 | 2.61 | 3.99 | 3.13 | 2.65 | 4.06 | 0.83 |
| secondorder | ShortRunHighGrayLevelEmphasis | 40.06 | 27.36 | 64.82 | 71.46 | 42.97 | 648.23 | 0.266 |
| secondorder | RunLengthNonUniformity | 30888.95 | 8539.46 | 68401.11 | 30051.53 | 9790.21 | 73898.76 | 0.692 |
| secondorder | ShortRunEmphasis | 0.74 | 0.7 | 0.78 | 0.74 | 0.7 | 0.77 | 0.811 |
| secondorder | LongRunHighGrayLevelEmphasis | 170.86 | 100.12 | 299.46 | 315.83 | 181.99 | 3784.55 | 0.227 |
| secondorder | RunPercentage | 0.67 | 0.61 | 0.71 | 0.67 | 0.61 | 0.71 | 0.892 |
| secondorder | LongRunLowGrayLevelEmphasis | 0.07 | 0.04 | 0.11 | 0.04 | 0 | 0.07 | 0.229 |
| secondorder | RunEntropy | 3.71 | 3.55 | 3.85 | 3.86 | 3.7 | 3.99 | 0.609 |
| secondorder | HighGrayLevelRunEmphasis | 56.05 | 37.65 | 86.63 | 96.89 | 59.94 | 890.69 | 0.25 |
| secondorder | RunLengthNonUniformityNormalized | 0.51 | 0.46 | 0.56 | 0.51 | 0.46 | 0.56 | 0.822 |
| secondorder | GrayLevelVariance | 5.3 | 4.35 | 6.23 | 7.55 | 5.13 | 10.58 | 0.234 |
| secondorder | ZoneVariance | 785163.19 | 199150.42 | 2434205.8 | 788364.99 | 140554.72 | 2317558.28 | 0.528 |
| secondorder | GrayLevelNonUniformityNormalized | 0.16 | 0.14 | 0.19 | 0.14 | 0.12 | 0.17 | 0.545 |
| secondorder | SizeZoneNonUniformityNormalized | 0.31 | 0.28 | 0.34 | 0.3 | 0.28 | 0.33 | 0.683 |
| secondorder | SizeZoneNonUniformity | 727.3 | 222.12 | 1875.99 | 752.64 | 255.63 | 1759.79 | 0.663 |
| secondorder | GrayLevelNonUniformity | 395.89 | 118.8 | 1017.12 | 294.74 | 130.38 | 1023.62 | 0.742 |
| secondorder | LargeAreaEmphasis | 785853.52 | 199809.46 | 2435144.03 | 789098.02 | 140935.37 | 2319334.9 | 0.529 |
| secondorder | SmallAreaHighGrayLevelEmphasis | 35.02 | 23.56 | 54.99 | 52.15 | 35.19 | 551.77 | 0.27 |
| secondorder | ZonePercentage | 0.03 | 0.02 | 0.04 | 0.03 | 0.02 | 0.04 | 0.84 |
| secondorder | LargeAreaLowGrayLevelEmphasis | 11144.83 | 2611.8 | 28865.88 | 3499.35 | 1380.75 | 12364.57 | 0.134 |
| secondorder | LargeAreaHighGrayLevelEmphasis | 44609055.9 | 10225005.4 | 150812256 | 95283216.3 | 14932378.2 | 713853494 | 0.758 |
| secondorder | HighGrayLevelZoneEmphasis | 62.05 | 41.34 | 92.7 | 94.28 | 64.15 | 881.14 | 0.271 |
| secondorder | SmallAreaEmphasis | 0.57 | 0.54 | 0.6 | 0.57 | 0.54 | 0.59 | 0.662 |
| secondorder | LowGrayLevelZoneEmphasis | 0.03 | 0.02 | 0.05 | 0.02 | 0 | 0.03 | 0.233 |
| secondorder | ZoneEntropy | 5.28 | 4.94 | 5.57 | 5.59 | 5.23 | 5.91 | 0.544 |
| secondorder | SmallAreaLowGrayLevelEmphasis | 0.02 | 0.01 | 0.03 | 0.01 | 0 | 0.02 | 0.266 |
| higherorder | Coarseness | 0 | 0 | 0 | 0 | 0 | 0 | 0.539 |
| higherorder | Complexity | 41.05 | 29.99 | 75.13 | 76.84 | 45.5 | 557.2 | 0.8 |
| higherorder | Strength | 0.01 | 0 | 0.03 | 0.04 | 0.01 | 0.16 | 0.159 |
| higherorder | Contrast | 0.01 | 0.01 | 0.02 | 0.01 | 0 | 0.02 | 0.62 |
| higherorder | Busyness | 52.15 | 23.4 | 161.04 | 20.16 | 9.96 | 56.43 | 0.343 |
